# A genome-wide association study of thyroid stimulating hormone and free thyroxine in Danish children and adolescents

**DOI:** 10.1371/journal.pone.0174204

**Published:** 2017-03-23

**Authors:** Tenna Ruest Haarmark Nielsen, Emil Vincent Rosenbaum Appel, Mathilde Svendstrup, Johanne Dam Ohrt, Maria Dahl, Cilius Esmann Fonvig, Mette Hollensted, Christian Theil Have, Haja N. Kadarmideen, Oluf Pedersen, Torben Hansen, Jens-Christian Holm, Niels Grarup

**Affiliations:** 1 The Children’s Obesity Clinic, Department of Pediatrics, Copenhagen University Hospital Holbæk, Holbæk, Denmark; 2 The Novo Nordisk Foundation Center for Basic Metabolic Research, Section of Metabolic Genetics, Faculty of Health and Medical Sciences, University of Copenhagen, Copenhagen, Denmark; 3 Danish Diabetes Academy, Odense, Denmark; 4 Department of Large Animal Sciences, Faculty of Health and Medical Sciences, University of Copenhagen, Frederiksberg C, Denmark; 5 Department of Bio and Health Informatics, Technical University of Denmark, Kgs. Lyngby, Denmark; 6 Department of Clinical Medicine, University of Copenhagen, Copenhagen, Denmark; McMaster University, CANADA

## Abstract

**Background:**

Hypothyroidism is associated with obesity, and thyroid hormones are involved in the regulation of body composition, including fat mass. Genome-wide association studies (GWAS) in adults have identified 19 and 6 loci associated with plasma concentrations of thyroid stimulating hormone (TSH) and free thyroxine (fT4), respectively.

**Objective:**

This study aimed to identify and characterize genetic variants associated with circulating TSH and fT4 in Danish children and adolescents and to examine whether these variants associate with obesity.

**Methods:**

Genome-wide association analyses of imputed genotype data with fasting plasma concentrations of TSH and fT4 from a population-based sample of Danish children, adolescents, and young adults, and a group of children, adolescents, and young adults with overweight and obesity were performed (N = 1,764, mean age = 12.0 years [range 2.5−24.7]). Replication was performed in additional comparable samples (N = 2,097, mean age = 11.8 years [1.2−22.8]). Meta-analyses, using linear additive fixed-effect models, were performed on the results of the discovery and replication analyses.

**Results:**

No novel loci associated with TSH or fT4 were identified. Four loci previously associated with TSH in adults were confirmed in this study population (*PDE10A* (rs2983511: *β* = 0.112*SD*, *p* = 4.8 ∙ 10^−16^), *FOXE1* (rs7847663: *β* = 0.223*SD*, *p* = 1.5 ∙ 10^−20^), *NR3C2* (rs9968300: *β* = 0.194*SD*), *p* = 2.4 ∙ 10^−11^), *VEGFA (*rs2396083: *β* = 0.088*SD*, *p* = 2.2 ∙ 10^−10^)). Effect sizes of variants known to associate with TSH or fT4 in adults showed a similar direction of effect in our cohort of children and adolescents, 11 of which were associated with TSH or fT4 in our study (*p*<0.0002). None of the TSH or fT4 associated SNPs were associated with obesity in our cohort, indicating no pleiotropic effects of these variants on obesity.

**Conclusion:**

In a group of Danish children and adolescents, four loci previously associated with plasma TSH concentrations in adults, were associated with plasma TSH concentrations in children, suggesting comparable genetic determinants of thyroid function in adults and children.

## Introduction

Thyroid hormone concentrations exhibit a physiological narrow variability within the individual, but exhibit a considerable inter-individual variability [1]. This could be explained by a genetic influence, which has been shown to explain 65% of the variation in concentrations of thyroid stimulating hormone (TSH) and thyroxine (T4) in healthy adults [2]. Nineteen loci have been found to be associated with circulating TSH and six with free T4 (fT4) concentrations in genome-wide association studies (GWAS) in adults [3–12]. Despite a tight physiological regulation between TSH and fT4, these two hormones do not seem to share genetic influences [13]. A recent study showed that all common variants could explain ~20% of the variation in TSH and fT4 concentrations [12]. However, the identified loci specific to TSH only explained a small part of the variation in TSH concentration, suggesting that a part of the missing heritability in relation to thyroid hormones might be explained by larger effects of rare or low-frequency variants [12]. Only a few GWAS on TSH and fT4 have included children [9,10]. Only one study in neonates (N = 1,583) has focused exclusively on children, replicating two associations previously identified in adults; one in *PDE8B* and one upstream of *FOXE1* [14]. The scarcity of studies in children makes it difficult to determine, whether the loci identified to be important for TSH and fT4 in adults, are also important during childhood and adolescence. Although associations with certain phenotypes, such as BMI and vitamin D, identified in adults have been replicated in children [15,16], results from GWAS of BMI in children and adolescents have also contributed with additional genetic associations [17]. Even during childhood the effects of genetic variants may act in opposite directions, as exemplified by a negative influence of some *FTO* variants in early childhood compared to a positive effect in later childhood [18], indicating that investigations in children may help explain more of the time-dependent influence of genetic variation on a given phenotype. The age-dependent effects of genetic variations on TSH and fT4 were illustrated in a recent study, in which a gene risk score (GRS) based on studies in adults explained considerably less of the variation in TSH and fT4 in newborns compared to children at six years of age (TSH 0.8−1.0%, fT4 0.2−0.3% vs. TSH 5.3−5.5%, fT4 1.9−3.6%) [19]. This difference may be biased by the maternal thyroid concentrations, which are known to pass the placental barrier. Thus the thyroid function of the newborn is not established and stabilized until several days after birth [19,20]. In adults, up to 7.1% of the variation in serum TSH concentrations and 1.9% of the variation in serum fT4 concentrations have been explained by gene risk scores for the given phenotype [12], thus there is still unexplained variation in children, not accounted for by the variation described in studies in adults.

Thyroid hormones are known to fluctuate during growth and development [21], and in adulthood, concentrations increase with age [22]. Higher concentrations of TSH and lower concentrations of fT4 have been associated with adiposity in both adults and children [23–28]. In addition, some variants associated with TSH have exhibited an attenuated effect on increasing body mass index (BMI) [4,29]. However, whether measures of thyroid function and adiposity have a shared genetic background is not clear. Variations in circulating concentrations of TSH and fT4, even within the normal range, have been associated with cardiovascular mortality, metabolic syndrome, dyslipidemia, and depression, illustrating the diverse effects of the thyroid axis [30].

The aim of the present study was to identify genetic variants associated with concentrations of TSH and fT4 in a cohort of Danish children and adolescents with normal weight and overweight/obesity, and to elucidate possible effects on obesity of these associations.

## Patients and methods

### Study population

A total of 1,876 children, adolescents, and young adults aged 2−25 years with obesity were recruited from The Children’s Obesity Clinic, Department of Pediatrics, Copenhagen University Hospital Holbæk, at entry into childhood obesity treatment [31]. The only criteria for inclusion into treatment was a BMI above the 90^th^ percentile for age and sex according to a Danish reference population [32]. There were no exclusion criteria for entering into treatment.

As part of The Danish Childhood Obesity Biobank (ClinicalTrials.gov ID-no.: NCT00928473), a population-based group of 2,555 children and adolescents was recruited from local schools, where all students between 6 and 22 years of age were invited to participate.

The only exclusion criterion for the present study was an ethnicity other than Danish/North-European white (N = 365).

GWAS analyses, with TSH and fT4 as phenotypes, were performed on the discovery batch comprising 1,900 individuals included from January 2009 to March 2013. The replication batch consisted of 2,166 individuals recruited from March 2013 to May 2015. The same exclusion criterion applied to both batches.

All participants were examined at baseline with anthropometrics and fasting blood samples (S1 Table).

All participants received oral and written information and gave informed assent. Informed written consent was obtained from parents/caretakers of children younger than 18 years of age, and from participants 18 years of age and older. The study was approved by the Danish Data Protection Agency, the Regional Ethics Committee of Region Zealand (SJ-104), and was performed according to the Helsinki Declaration.

### Objective clinical measurements

#### Anthropometrics

Height was measured by stadiometer to the nearest 0.1 cm. Weight was measured to the nearest 0.1 kg. Measurements were performed without shoes and with the child wearing light indoor clothes. BMI was calculated as weight in kilograms divided by height in meters squared, and a BMI standard deviation score (SDS) was calculated according to the LMS method [33] based on Danish reference charts [32].

#### Blood samples

Blood samples were drawn from the antecubital vein after an overnight fast. Plasma concentrations of TSH and fT4 were measured on a Cobas 6000® analyzer (Roche Diagnostics, Manheim, Germany) and on a Dimension Vista (Siemens Healthcare, Erlangen, Germany) in the discovery and replication batch, respectively. Both devices use immunologic chemiluminescent analysis methods. Intra-individual coefficients of variation on the Cobas 6000® were 1.4−2.4% for TSH and 1.7−2.6% for fT4, and on the Dimension Vista 1.3−1.4% for TSH and 1.8−1.9% for fT4.

### Genetic analyses

#### Genotyping and imputation

DNA was extracted at LGC Genomics (LGC, Middlesex, United Kingdom). All participants were genotyped with the Illumina Infinium Human CoreExome BeadChip (CoreExomeChip) using Illumina’s HiScan system at the Novo Nordisk Foundation Centre for Basic Metabolic Research’s laboratory at Symbion, Copenhagen, Denmark. The standard pipeline in Illumina Genome Studio software was used for the genotype calling. A total of 503,035 markers in 1,900 individuals entered the quality control (QC) pipeline. Due to a call-rate below 95%, 317 markers were removed. Furthermore, 129 individuals were excluded due to 1) a call-rate below 95%, 2) extreme positive or negative inbreeding coefficients, 3) ethnic outliers using Principal Component Analysis (PCA) on ancestral markers, 4) unknown first degree relative relations found by Identical By Descent (IBD) analysis where only the relative with the highest call-rate for each pedigree-pair was retained, 5) sample duplicates, and 6) sex discrepancy between genotype and phenotype data. In our QC pipeline, we used a combination of scripts written in Python and R[34] together with PLINK software [35]. This left 1,771 individuals with 502,718 markers available for further analyses.

Haplotypes were phased using SHAPEIT [36] and additional genotypes were imputed by IMPUTE2, applying the 1000 genomes phase 1 reference panel [37] on all markers that passed a Hardy-Weinberg Equilibrium (HWE) filter (*p*<0.005), to a final of ~16.8 million markers. Markers with a minor allele frequency (MAF) below 1%, an imputation quality below 0.6, or not in HWE (*p*<1 ∙ 10^−6^) after imputation, were excluded (a total of ~8.3 million markers for combined QC criteria), leaving ~8.5 million markers for the final analyses. After QC and imputation 91 individuals were removed due to missing phenotype data, making the final number for the discovery analysis of both TSH and fT4 1,680 (S1 Fig).

In the replication study, six lead SNPs in 2,166 individuals were genotyped with KASP SNP Assay (LGC Genomics, Middlesex, UK), while the remaining three lead SNPs failed direct genotyping. Instead, proxy SNPs were genotyped, using the same platform. Proxies were selected based on 1) SNP present on the CoreExome chip, to assure well designed assays had been successfully made earlier, 2) Strongest association to TSH/fT4, and 3) Minimal distance to the SNP from the discovery analyses which failed to replicate (S2 Table). For rs5997852 no suitable proxy could be identified using the criteria defined above. Therefore, we identified other possible proxies for rs5997852 in our discovery cohort. Sixteen SNPs were in high LD (R^2^>0.75). rs28360515 showed the highest LD with rs5997852, and was thus selected for replication (S2 Table). Prior to genotyping the LD was validated in adults in the Inter99 cohort [38].

In the replication cohort, individuals with self-reported ethnicity other than Danish/North-European white (N = 37) were removed. In total, 2,129 individuals passed QC with a call-rate of at least 99% for each of the six SNPs, without using any cut-offs on call-rate. See S1 Table for basic characteristics of participants included in the analyses.

### Statistical analyses

The discovery association analyses were performed using an additive linear model implemented in the analytical software SNPTEST (v2) [36], using a frequentist score-test to characterize statistical uncertainty [39]. TSH and fT4 concentrations were transformed to a standard normal distribution, to avoid phenotypic outliers to skew results, using a rank based inverse normal transformation. S3 Table shows the skewness and kurtosis for the two phenotypes before transformation. We identified 11 outliers for TSH and 31 outliers for fT4 (6 were outliers for both TSH and fT4). Outliers were defined as TSH<0.45 mIU/L or >10 mIU/L, or fT4<11.1 pmol/L or >23.4 pmol/L according to references from pediatric populations and the cut-off for subclinical hypothyroidism [20,40,41]. All models were adjusted for sex and age. Additionally, we ran another discovery analysis, adjusted for BMI SDS. We calculated principal components based on uncorrelated variants. However, since the principal components did not associate with the phenotypes and ethnic outliers were removed in the genotyping QC, we did not adjust for principal components in the discovery analysis.

The *p*-values were adjusted using the method of genomic control (GC) by calculating the inflation factor (*λ*) of the *p*-values as $\lambda =\frac{\text{median}(\chi_{1}^{2}(1-P))}{\text{median}(\chi_{1}^{2}(0.5))}$, where $\chi_{1}^{2}$ is the (non-central) chi-squared distribution with one degree of freedom and *p* is the observed *p*-values in the discovery analyses. The adjusted *p*-values were then calculated as: $p_{adjusted}=p_{\chi_{1}^{2}}\left(\frac{\chi_{1}^{2}(1-p)}{\lambda }\right)$, where $p_{\chi_{1}^{2}}$ is the probability distribution for a non-central chi-squared distribution as implemented in the R-function chi-square from the R-packages *stat* [42].

In the discovery analysis, a replication threshold of *p* < 1 ∙ 10^−6^ was used in the identification of loci with potential associations with TSH or fT4. To test whether the SNPs reaching the replication threshold were previously known or potentially novel, we performed a conditioned analysis on each of our lead SNPs, defined as the strongest signal in the locus, dependent on SNPs on the same chromosome and known to associate with the specific phenotype (S4 Table) [3–12].

### Meta-analysis

The results from the discovery and replication stages were combined in a fixed-effect meta-analysis, with alleles flipped to match the effect allele from the discovery analysis. We report the *p*-values for the two-tailed test on the combined effect. A genome-wide significant threshold of *p* < 5 ∙ 10^−8^ was applied for these analyses. We used the R-package ‘meta’ for these analyses.

### Weighted gene risk score (wGRS)

We used the strongest signal in each locus, known to associate with TSH or fT4 concentrations in adults, and calculated a weighted gene risk score (wGRS), weighted by the effect per allele as identified in our discovery analysis. The effect allele in each locus was chosen as the allele that increased the TSH or fT4 concentrations. The wGRS was then used as the explanatory variable in an additive linear model on rank normalized TSH and fT4, with age and sex as co-variables. To estimate how much of the variation was captured by the wGRS, we used ANOVA (analysis-of-variance, type-II) from the *car* package [43] in R to calculate the sum of squares proportion for the wGRS. The 95% confidence interval (*CI*_95%_) for this estimation was calculated using the standard approach for computing the confidence interval for proportions, by using the formula in R: ${CI}_{95\%}=\frac{k}{N}\pm qnorm(0.975)∙\sqrt{\frac{\frac{k}{N}∙(1-\frac{k}{N})}{N}}$, where k is the sum of squares proportion for the wGRS, N is the sample size, and qnorm (0.975) is the 97.5^th^ percentile of the normal distribution.

### Associations with obesity and BMI SDS

The associations between obesity, defined as a BMI SDS above 1.28, corresponding to the 90^th^ percentile for age and sex [32], and the lead SNPs, significantly associated with TSH or fT4 concentrations in the meta-analyses, were investigated. Analyses were performed using a logistic regression model and a case-control design, adjusted for sex and age at baseline. A subgroup of lean participants from the population-based group, with -1.28 ≤BMI SDS≤1.28 [32] (N = 1,788), was compared to a subgroup with obesity combining the group of children and adolescents from The Children’s Obesity Clinic with the children and adolescents from the population-based sample, who had a BMI SDS >1.28 [32] (N = 2,397). As we investigated whether the selected lead SNPs, significantly associated to TSH or fT4 concentrations, also associated with obesity, adjusted for sex and age, or with BMI SDS as a continuous variable, using a fixed-effect meta-analysis, a significance threshold adjusted for multiple testing *ad modum* Bonferroni of *p* < 0.0056 was chosen for these analyses. We used the R-package ‘meta’ for these analyses.

### Additional analyses of the VEGFA locus

We investigated associations of two SNPs (rs2396083 and rs6905288) in the *VEGFA* locus to the inverse rank normalized waist hip ratio (WHR) in a linear additive model, adjusted for age and sex, as SNPs in the VEGFA locus have been associated with WHR in European adults [44]. We also investigated the LD between the two variants using the online tool LDlink [45] in Europeans from phase 3 of the 1000 Genome Project.

### Statistical power

We calculated the statistical power to detect signals above a certain threshold, given a specific MAF and effect size, by running a linear additive model on a simulated normal distribution with the same variation as the trait in our study. We then observed how often the model resulted in a significant test at a given significance level. For each effect size and MAF, we performed the simulation 100 times (S5 Table).

## Results

**Fig 1:**
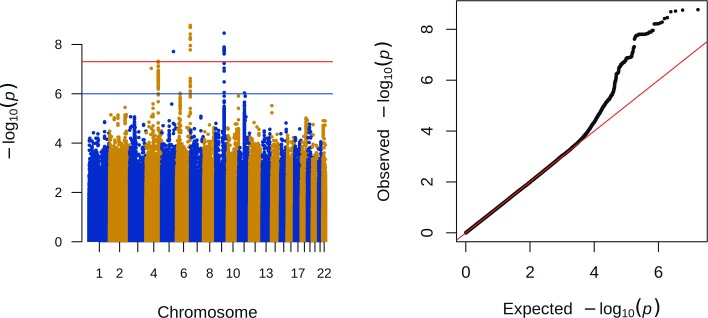
Association of genome-wide variants with plasma TSH concentrations. SNPs are plotted on the x-axis according to their chromosomal position against the–log10(*p*-value). The results were considered genome-wide significant with a *p*<5·10^−8^. A threshold for replication was set at *p*<1·10^−6^.

A total of 1,680 individuals were available for the TSH discovery analysis of 8,508,717 genetic markers. Of these, 183 markers in seven loci reached the replication threshold of *p* < 1 ∙ 10^−6^ in the discovery analyses (Table 1 and Fig 1). When we adjusted for BMI SDS, we observed comparable results for the seven identified loci and no additional associated loci were detected. We performed the analyses stratified for sex, which did not reveal additional associated loci (data not shown).

**Table 1:** Lead SNPs from the discovery-, replication- and meta-analyses.

	**Info**		**GWAS results**				**Replication**				**Meta-analysis**		
**SNP**	Locus	Effect/Non effect allele	EAF	Beta	SE	P	EAF	Beta	SE	P	Beta	SE	P
**TSH**													
**rs2983511**	*PDE10A*	G/C	0.696	0.238	0.038	1.7·10^−9^	0.605	0.093	0.015	3.4·10^−10^	0.112	0.014	4.8·10^−16^
rs7847663*	*FOXE1*	T/C	0.679	0.226	0.037	3.5·10^−9^	0.698	0.220	0.031	2.8·10^−12^	0.223	0.024	1.5·10^−20^
**rs77601015**	*HMHB1*	T/C	0.983	1.029	0.178	1.9·10^−8^	0.968	-0.198	0.125	1.3·10^−1^	0.207	0.102	4.3·10^−2^
**rs9968300**	*NR3C2*	C/A	0.809	0.253	0.045	4.8·10^−8^	0.795	0.152	0.038	6.7·10^−5^	0.194	0.029	2.4·10^−11^
**rs74781923**	*TECRL*	C/T	0.989	1.161	0.211	9.3·10^−8^	0.970	-0.117	0.184	5.3·10^−1^	0.435	0.139	1.7·10^−3^
rs75732991*	*FAM86C1*	G/A	0.511	0.188	0.037	9.3·10^−7^	0.588	0.011	0.030	7.2·10^−1^	0.079	0.023	6.2·10^−4^
**rs2396083**	*VEGFA*	C/G	0.714	0.200	0.040	9.5·10^−7^	0.593	0.072	0.015	1.1·10^−6^	0.088	0.014	2.2·10^−10^
**fT4**													
rs5997852*	*SMTN*	G/A	0.483	0.204	0.039	3.4·10^−7^	0.481	0.013	0.024	5.9·10^−1^	0.063	0.020	1.9·10^−3^
**rs144590826**	*FAM205B*	T/C	0.012	0.863	0.168	4.0·10^−7^	0.025	0.001	0.004	7.9·10^−1^	0.002	0.014	6.7·10^−1^

SNPs are listed according to their association with each of the traits of fasting plasma TSH and fT4 concentrations.

* Indicates a proxy was used for the replication (S6 Table).

Of the seven identified loci, four have previously been associated with variations in circulating TSH concentrations. The remaining three lead SNPs (rs77601015, rs74781923, rs75732991) were considered markers of potentially novel loci, as they maintained their significance level after conditioning on known TSH-associated SNPs (Table 1 and S4 Table).

We sought to replicate the seven identified lead SNPs associated with TSH concentrations. Three of the seven lead SNPs identified in the discovery analyses were significantly associated (*p*<0.0056) with TSH concentrations in the replication sample (rs2983511, rs2396083, and rs9968300). Two lead SNPs (rs7847663 and rs75732991) were unsuccessfully genotyped; instead proxies were identified and genotyped. In the replication batch the proxy SNP (rs925489) for rs7847663 associated with TSH (p<0.0056) (Table 1). The remaining SNPs were not significantly associated with TSH concentrations in the replication study (Table 1).

From the discovery and replication sample sets, 3,809 individuals with TSH measurements were available for meta-analyses. The four SNPs associating significantly with TSH concentrations in the discovery and replication analyses all reached a genome-wide significance (*p*<5·10^−8^) in the meta-analyses (Table 1). The four identified lead SNPs were all located near loci previously known to associate with TSH in adults [3].

**Fig 2:**
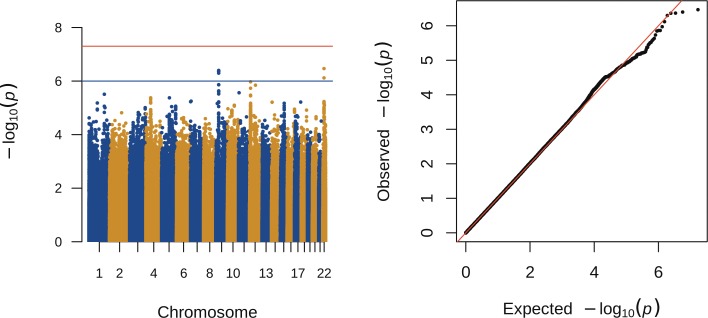
Association of genome-wide variants with plasma fT4 concentrations. SNPs that passed QC are plotted on the x-axis according to their chromosomal position against their–log10(*p*-value). The results were considered genome-wide significant with a *p*<5·10^−8^ and a replication threshold was set at *p*<1·10^−6^.

A total of 1,680 individuals were available for the fT4 analyses. In the discovery analyses, six markers in two potentially novel loci reached the replication threshold of *p* < 1 ∙ 10^−6^ (Table 1 and Fig 2).

We attempted to replicate the two identified loci. In the replication sample, rs5997852 could not be successfully genotyped, so a proxy was chosen for rs5997852. Neither this proxy (rs28360515) or rs144590826 associated with fT4 in 2,129 individuals from the replication batch, nor did they associate at genome-wide significance in the meta-analysis of discovery and replication studies (Table 1).

**Fig 3:**
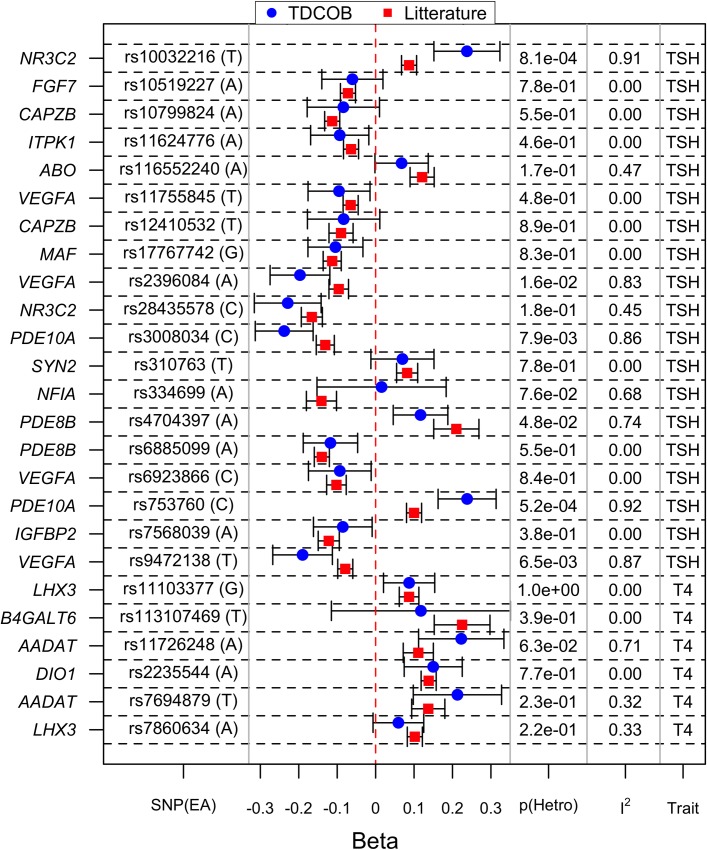
Effects of genetic variants known to associate with plasma TSH or fT4 concentrations. Effects from studies in adults compared to the effects of the variants in the cohort from the Danish Childhood Obesity Biobank (TDCOB). Effect sizes are shown in standard deviations (SD) of the rank-normalized TSH or fT4 distribution with 95% confidence intervals. EA is the Effect Allele (from the literature). I^2^ is the measure for heterogeneity between the TDCOB cohort and literature. p(Hetro) is the p-value for the heterogeneity.

The reported effect sizes of known loci on the TSH and fT4 concentrations in adults [3–12] were compared to the effect sizes in our cohort of children and adolescents (Fig 3 and S6 Table). For all loci, the effects on TSH and fT4 in our cohort were in the same direction as previously described in the literature in adults. In our study, 17 of 25 loci were nominally significantly associated with TSH or fT4 (*p*<0.05), and 11 remained significant when correcting for multiple testing *ad modum* Bonferroni (*p*<0.0002) (S6 Table). For two SNPs in two loci (rs10032216 in *NR3C2* and rs753760 in *PDE10A*) the heterogeneity was above 90% and statistically significant after Bonferonni correction (p < 0.002). The effect size was greater in our cohort compared to the observe effect in the literature (Fig 3).

The wGRS of SNPs previously known to associate with TSH concentrations also associated with TSH concentration in the present study (β = 0.053, SE = 0.015, *P* = 3.2·10^−4^), but not with fT4 concentrations (β = 0.006, SE = 0.015, *P* = 0.19). Similarly, the wGRS for fT4 associated positively with fT4 concentrations (β = 0.025, SE = 0.004, *P* = 7.5·10^−9^) in our cohort but not with TSH concentrations (β = -0.011, SE = 0.004, *P* = 0.45). The wGRS for TSH explained 0.5% of the variation in TSH concentrations. The wGRS for fT4 explained 1.8% of the variation in fT4 concentrations. In participants 12 years of age or younger (N = 871), the wGRS explained 0.6%(*CI*_95%_[0.083%-1.1%]) of the variation in TSH concentrations and 3.7%(*CI*_95%_[2.5%-5%]) of the variation in fT4 concentrations. In participants older than 12 years of age (N = 816), the wGRS explained 0.1%(*CI*_95%_[0%-0.3%]) of the variation in TSH concentrations and 0.8%(*CI*_95%_[0.2%-1.4%]) of the variation in fT4 concentrations.

Applying a 5% significance threshold, no associations were found between the four lead SNPs associated with TSH and fT4 concentrations and obesity, defined as a BMI SDS >1.28 or BMI SDS as a continuous variable, when combining 3,894 individuals (1,497/2,397 obese/lean) from both the discovery and replication sample sets in a meta analysis (S7 Table and S2A–S2D Fig).

We investigated two variants in the *VEGFA* locus; our lead SNP: rs2396083 and the variant rs6905288, which have been found to associate with WHR [44]. We saw no LD between the two variants in the European population (D' = 0.0024, R^2^ = 0.0 using LDlink [45]), and found no association with WHR for either variant in our discovery set (*P*_*rs*2396083_ = 0.42, *P*_*rs*6905288_ = 0.29). Further adjusting for BMI SDS in this analysis did not alter this result (data not shown).

## Discussion

In a GWAS of 1,680 children and adolescents and replication in an additional set of 2,129 children and adolescents, four SNPs reached genome-wide significance for their associations with TSH concentrations (*PDE10A* (rs2983511), *FOXE1* (rs7847663), *NR3C2* (rs9968300), *VEGFA* (rs2396083)). These four loci have all previously been associated with serum TSH concentrations in adults [3].

From the literature we identified 32 SNPs in 19 loci previously found to associate with TSH or fT4 concentrations in adults [3–12], 25 of which were present in our study. When comparing effect sizes of 25 of the 32 SNPs in our cohort of children and adolescents with effects described in the literature, the same directional effects were observed with predominantly overlapping *CI*_95%_. The *NR3C2* locus and one of the SNPs in the *PDE10A* locus (rs753760) seemed to have a larger effect in our cohort. However, as we compared beta-values between studies, each on their own rank normalized scale, we cannot prove any actual difference in effect. The heterogeneity between our cohort and the cohort from the literature was >90% for both loci. This difference may be genuinely due to different effects in adults and children, but could also be due to gene-gene or gene-environment interactions. In comparison, among the previous studies of children, one cohort included children as young as 8 years of age, however, the combined meta-analysis consisted mostly of adults [3]. The *NR3C2* locus has been implicated in the insulin-like growth factor’s effects on thyroid function, and the difference could be explained by fluctuating concentrations of insulin-like growth factor during growth and development in childhood, before settling at a lower level in adulthood [3,46].

From the SNPs previously found to associate with either TSH or fT4 concentrations, two wGRSs were computed. These wGRSs were positively associated with TSH and fT4 concentrations in our cohort. The wGRSs were specific to either TSH or fT4 concentrations, i.e. the GRS for fT4 did not associate with TSH concentrations and vice versa. This is in line with twin studies of the heritability of thyroid variables showing that levels of both TSH and fT4 are heritable, but do not share genetic influence [13]. In our cohort, the wGRS explained 0.5% of the variation in TSH concentrations and 1.8% of the variation in fT4 concentrations. A larger degree of the variation in both TSH and fT4 concentrations tended to be explained by the wGRS in younger age (<12 years of age). Compared to studies in adults, the wGRS explained less of the variation in plasma TSH concentrations and, in children older than 12 years, less of the variation in fT4 concentrations [12]. The wGRS in the youngest part of our cohort also explained considerably less of the variation in TSH concentrations compared to what has been found in a group of 6-year-old children [19].

Variations in TSH and fT4 concentrations, even within the normal range, have been associated with variations in BMI and body composition in both children and adults [13,28,27]. In a study by Malinowski *et al*. in 4,501 European American individuals, three SNPs in two loci associated with TSH (*NFIA* (rs10489909), *NRG1* (rs2466067, rs4298457)) were also associated with BMI in adults [4]. We did not find any association between our lead SNPs and childhood obesity or BMI SDS as a continuous variable. This is in line with another study in 867 children with obesity [29], investigating the effects of variations in *PDE8B* on TSH concentrations, which is strongly expressed in the thyroid gland [47], finding these effects to be independent of BMI, but with an additive effect of BMI on TSH concentrations [29]. The three SNPs reported to be associated with BMI in adults [4] did not associate with plasma TSH concentrations in our cohort.

One of the loci, *VEGFA*, in which one of our lead SNPs is located, has previously been associated with WHR, which is a marker of abdominal adiposity, however, in line with our study, they did not find any association to BMI [44]. We did not find any association between two SNPs in this locus and WHR in our study. We did not find any published GWAS implicating the *PDE10A*, *FOXE1*, or *NR3C2* loci in obesity related diseases. Thus our results suggest, that the associations between TSH and the *PDE10A*, *FOXE1*, and *NR3C* loci could be independent of BMI SDS.

The strengths of the present study were a group of ethnically homogeneous Danish/North-European White children, with a prospectively collected cohort, which enabled replication in another part of our own cohort. Finally, the construction of the cohort with lean controls and cases with obesity enabled us to estimate the influence of the TSH associated SNPs on childhood obesity. A thorough questionnaire provided comprehensive information on medications and other diseases enabling exclusions of individuals with secondary changes in thyroid function. We performed sensitivity analyses excluding individuals with known thyroid disease, individuals taking medications known or suspected to influence thyroid function [48], and individuals with known or suspected syndromes or monogenic obesity (N = 43), which did not change the results. We therefore proceeded with reporting on the entire cohort.

We found three possible novel variants associating with TSH in the discovery phase, but lacked statistical power (S5 Table) to determine if these findings were true. We sought to overcome this obstacle by replicating in another batch of the cohort, but were unsuccessful in finding any novel associations. Consequently, we were only able to replicate previously found loci from studies in adults. We did not have enough statistical power to find loci with smaller effect sizes identified in larger studies in adults, but we do generally observe similar effects in children for these loci. We were unable to identify any associations acting differently in our cohort of children and adolescents than what has been reported in adults.

In conclusion, *PDE10A* (rs2983511), *NR3C2* (rs9968300), *FOXE1* (rs7847663), and *VEGFA* (rs2396083), which have all previously been associated with TSH concentrations in adults, associated with TSH concentrations in a group of children and adolescents. The four identified loci showed similar effects on TSH concentrations as previously reported in adults, suggesting comparable genetic determinants of thyroid function in adults and children.

## Supporting information

S1 Fig Flowchart showing the number of participants in study, from recruitment to meta-analysis. (TIF)

S2 Fig Forest plots for meta-analyses of associations between rs2983511 and BMI SDS. (PNG)

S3 Fig Forest plots for meta-analyses of associations between rs9968300 and BMI SDS. (PNG)

S4 Fig Forest plots for meta-analyses of associations between rs2396083 and BMI SDS. (PNG)

S5 Fig Forest plots for meta-analyses of associations between rs925489 (proxy for rs7847663) and BMI SDS. (PNG)

S1 Table Descriptive information on the discovery and replication batches. (DOCX)

S2 Table Information on the proxy SNPs chosen for lead SNPs not able to be successfully genotyped in initial replication batch. Distance is measured in base-pairs (BP) between lead and proxy SNP. P(TSH) and P(fT4) are the unadjusted p-values from the discovery analysis.
(DOCX)

S3 Table Skewness and kurtosis for TSH and fT4 in the two sets (GWAS and replication) before transformation, showing that none of the phenotypes are normally distributed. (DOCX)

S4 Table Analyses of lead SNPs when conditioned on SNPs known to associate with the given trait (TSH or fT4) on the same chromosome. P (GC adjusted) is the *p*-value adjusted for genomic control in the discovery analysis. P (conditioned on known signals) is the *p*-value when the analysis is conditioned on the genotype for each SNP, on the same chromosome as the lead SNP, known to associate with the given trait.
(DOCX)

S5 Table Power table showing the minimum effect size (in SD units to obtain 80% statistically power (Minimum beta (SD) for 80% power) for a given MAF (0.05, 0.1, 0.2, or 0.45) after 100 simulations. The power to detect signals that crossed the replication threshold of *p*<1·10^−6^ in the discovery analysis (N = 1,680), and the power to detect signals with a p-value under the GWAS significance threshold of *p*<5·10^−8^ in the meta-analysis (N = 4,224), are given. The power to detect the odds ratio (OR) of overweight is calculated with a prevalence of obesity of 20% among children (Larsen LM, Hertel NT, Mølgaard C, Christensen R dePont, Husby S, Jarbøl DE. Prevalence of overweight and obesity in Danish preschool children over a 10-year period: a study of two birth cohorts in general practice. Acta Paediatrica. 2012 Feb;101(2):201–7). The discovery analysis consisted of 545/1123 cases/controls and the meta- analysis consisted of 1788/1932 cases/controls.
(DOCX)

S6 Table Overview of effects of known SNPs in adults and the effects identified in the discovery batch, along with the p-values from our discovery analysis. (DOCX)

S7 Table Validated lead SNPs or their proxy’s association to obesity and BMI SDS. (DOCX)

## Abbreviations

BMI: Body mass index
fT4: Free thyroxine
GC: Genomic control
HWE: Hardy-Weinberg Equilibrium
IBD: Identical By Descent
MAF: Minor allele frequency
PCA: Principal Component Analysis
QC: Quality control
SNP: Single nucleotide polymorphism
SD: Standard deviation
SDS: Standard deviation score
TSH: Thyroid stimulating hormone
WGRS: Weighted Gene Risk Score
