# Supplementary figures and images for: A genome-wide association study of thyroid stimulating hormone and free thyroxine in Danish children and adolescents
Source: PLoS One. 2017 Mar 23;12(3):e0174204. doi: 10.1371/journal.pone.0174204 (PMC5363901; doi:10.1371/journal.pone.0174204)

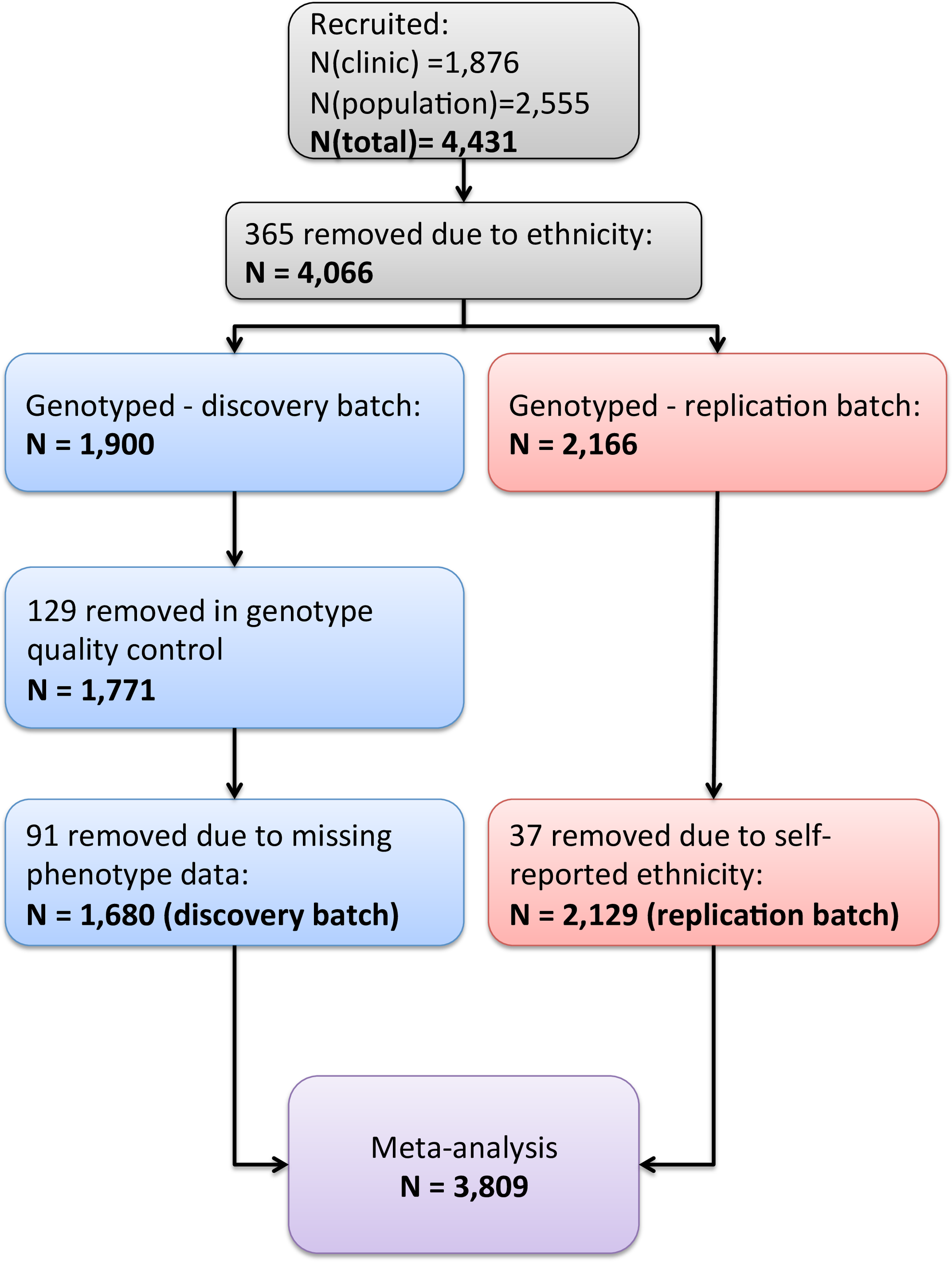

Supplement: S1 Fig — (TIF) [file pone.0174204.s001.tif]

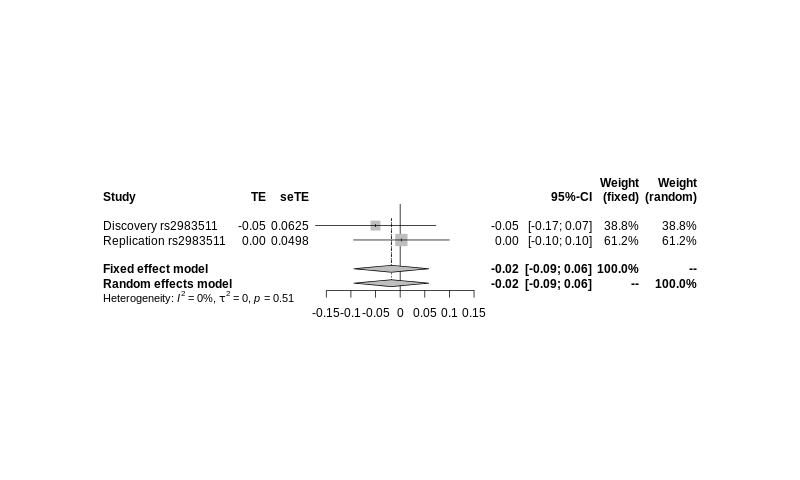

Supplement: S2 Fig — (PNG) [file pone.0174204.s002.png]

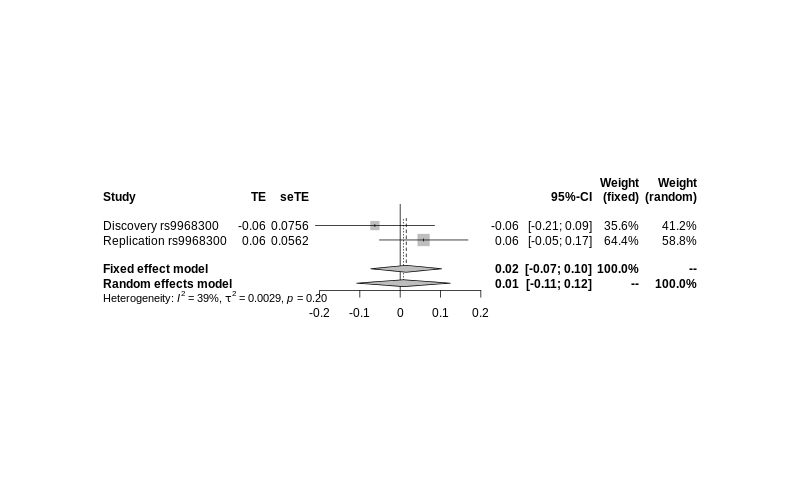

Supplement: S3 Fig — (PNG) [file pone.0174204.s003.png]

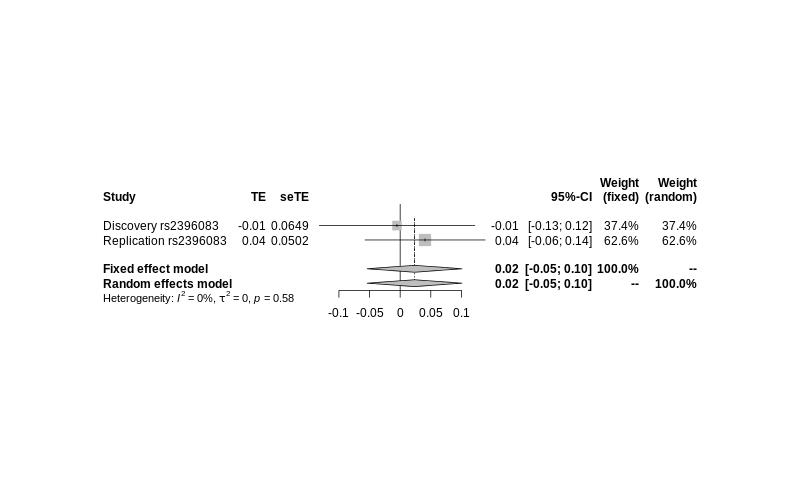

Supplement: S4 Fig — (PNG) [file pone.0174204.s004.png]

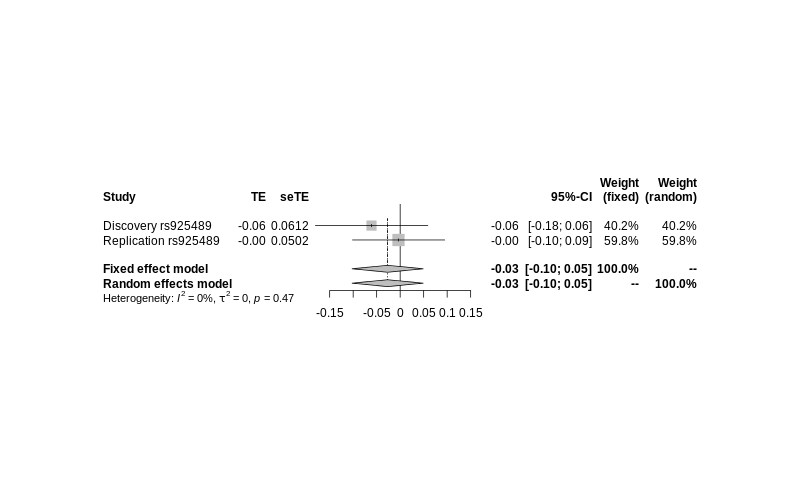

Supplement: S5 Fig — (PNG) [file pone.0174204.s005.png]
