# Supplementary material for: A genome-wide association study of thyroid stimulating hormone and free thyroxine in Danish children and adolescents
Source: PLoS One. 2017 Mar 23;12(3):e0174204. doi: 10.1371/journal.pone.0174204 (PMC5363901; doi:10.1371/journal.pone.0174204)
Supplement: S1 Table — (DOCX) [file pone.0174204.s006.docx]

| **Sample characteristics** | | | | |
| --- | --- | --- | --- | --- |
| Batch name | **Discovery** | **Replication** | **Overweight/Obese** | **Lean** |
| Sex (M/F) | 733/1002 | 864/1233 | 862/1070 | 699/1089 |
| CC (lean/obese) | 547/1162 | 1241/770 | 0/1932 | 1788/0 |
| Age (SD) | 11.8 (3.34) | 11.8 (3.49) | 11.6 (3.22) | 12.1 (3.62) |
| Age range | 2.47–24.73 | 1.20–22.80 | 1.20–24.70 | 5.60–22.80 |
| TSH (SD) | 2.67 (2.42) | 2.2 (1.79) | 2.55 (2.54) | 2.3 (1.32) |
| TSH range | 0.05–65.69 | 0.39–54.00 | 0.05–65.69 | 0.50–30.00 |
| fT4 (SD) | 15.4 (2.10) | 12.9 (2.18) | 14.2 (2.23) | 13.5 (2.65) |
| fT4 range | 8.40–36.30 | 1.80–70.8 | 1.80–36.3 | 8.20–70.8 |
| BMI SDS (SD) | 2.42 (1.50) | 0.68 (1.49) | 2.67 (0.78) | 0.11 (0.65) |
| BMI SDS range | -3.16–6.12 | -16.83–6.65 | 1.28–6.65 | -1.27–1.28 |
