# Supplementary material for: A genome-wide association study of thyroid stimulating hormone and free thyroxine in Danish children and adolescents
Source: PLoS One. 2017 Mar 23;12(3):e0174204. doi: 10.1371/journal.pone.0174204 (PMC5363901; doi:10.1371/journal.pone.0174204)
Supplement: S2 Table — Distance is measured in base-pairs (BP) between lead and proxy SNP. P(TSH) and P(fT4) are the unadjusted p-values from the discovery analysis. (DOCX) [file pone.0174204.s007.docx]

| **SNP** | **Chr** | **Position** | **Major/Minor Allele** | **MAF** | **Distance (BP)** | **R^2^** | **Proxy To** | **P(TSH)** | **P(fT4)** |
| --- | --- | --- | --- | --- | --- | --- | --- | --- | --- |
| rs925489 | 9 | 100546600 | C/T | 0.34 | 1732 | 1.00 | rs7847663 | 1.57E-08 | 2.71E-01 |
| rs2040301 | 11 | 71496121 | C/T | 0.43 | 3085 | 1.00 | rs75732991 | 1.44E-05 | 1.06E-03 |
| rs28360515 | 22 | 31436608 | C/T | 0.48 | 1973 | 0.92 | rs5997852 | NA | 6.56E-06 |
