# Supplementary material for: A genome-wide association study of thyroid stimulating hormone and free thyroxine in Danish children and adolescents
Source: PLoS One. 2017 Mar 23;12(3):e0174204. doi: 10.1371/journal.pone.0174204 (PMC5363901; doi:10.1371/journal.pone.0174204)
Supplement: S3 Table — (DOCX) [file pone.0174204.s008.docx]

|  | **GWAS** | | **Replication** | |
| --- | --- | --- | --- | --- |
|  | **TSH** | **fT4** | **TSH** | **fT4** |
| **Skewness** | 14.97 | 0.68 | 15.61 | 11.28 |
| **Kurtosis** | 363.73 | 6.00 | 402.95 | 273.18 |
