# Supplementary material for: A genome-wide association study of thyroid stimulating hormone and free thyroxine in Danish children and adolescents
Source: PLoS One. 2017 Mar 23;12(3):e0174204. doi: 10.1371/journal.pone.0174204 (PMC5363901; doi:10.1371/journal.pone.0174204)
Supplement: S4 Table — P (GC adjusted) is the p-value adjusted for genomic control in the discovery analysis. P (conditioned on known signals) is the p-value when the analysis is conditioned on the genotype for each SNP, on the same chromosome as the lead SNP, known to associate with the given trait. (DOCX) [file pone.0174204.s009.docx]

| **TSH** | | |
| --- | --- | --- |
| **SNP** | **P (GC adjusted)** | **P (conditioned on known signals)** |
| rs2983511 | 1.69·10^-9^ | 9.96·10^-1^ |
| rs7847663 | 3.47·10^-9^ | 7.47·10^-1^ |
| rs77601015 | 1.94·10^-8^ | 3.57·10^-9^ |
| rs9968300 | 4.83·10^-8^ | 3.33·10^-1^ |
| rs74781923 | 9.30·10^-8^ | 3.71·10^-8^ |
| rs75732991 | 9.28·10^-7^ | 3.77·10^-7^ |
| rs2396083 | 9.53·10^-7^ | 9.47·10^-1^ |
| **fT4** | | |
| **SNP** | **P (GC adjusted)** | **P (conditioned on known signals)** |
| rs5997852 | 3.42·10^-7^ | NA |
| rs144590826 | 4.00·10^-7^ | 1.11·10^-6^ |
