# Supplementary material for: A genome-wide association study of thyroid stimulating hormone and free thyroxine in Danish children and adolescents
Source: PLoS One. 2017 Mar 23;12(3):e0174204. doi: 10.1371/journal.pone.0174204 (PMC5363901; doi:10.1371/journal.pone.0174204)
Supplement: S5 Table — The power to detect signals that crossed the replication threshold of p<1·10−6 in the discovery analysis (N = 1,680), and the power to detect signals with a p-value under the GWAS significance threshold of p<5·10−8 in the meta-analysis (N = 4,224), are given. The power to detect the odds ratio (OR) of overweight is calculated with a prevalence of obesity of 20% among children (Larsen LM, Hertel NT, Mølgaard C, Christensen R dePont, Husby S, Jarbøl DE. Prevalence of overweight and obesity in Danish preschool children over a 10-year period: a study of two birth cohorts in general practice. Acta Paediatrica. 2012 Feb;101(2):201–7). The discovery analysis consisted of 545/1123 cases/controls and the meta- analysis consisted of 1788/1932 cases/controls. (DOCX) [file pone.0174204.s010.docx]

|  | **TSH** | | **fT4** | | **BMI SDS** | | **Overweight** | | |
| --- | --- | --- | --- | --- | --- | --- | --- | --- | --- |
|  | **Minimum beta (SD) for 80% power** | | **Minimum beta (SD) for 80% power** | | **Minimum beta (SD) for 80% power** | | **Minimum OR for 80% power** | | |
| **MAF** | **Discovery**  **(p<1·10^-6^)** | **Meta**  **(p<5·10^-8^)** | **Discovery**  **(p<1·10^-6^)** | **Meta**  **(p<5·10^-8^)** | **Discovery**  **(p<1·10^-6^)** | **Meta**  **(p<5·10^-8^)** | **Discovery**  **(p<1·10^-6^)** | | **Meta**  **(p<5·10^-8^)** |
| 0.05 | 1.18 | 0.61 | 1.01 | 0.77 | 0.69 | 0.51 | 2.22 | 1.81 | |
| 0.10 | 0.85 | 0.45 | 0.7 | 0.57 | 0.51 | 0.38 | 1.85 | 1.55 | |
| 0.20 | 0.66 | 0.33 | 0.51 | 0.41 | 0.37 | 0.27 | 1.61 | 1.42 | |
| 0.45 | 0.54 | 0.27 | 0.43 | 0.33 | 0.31 | 0.23 | 1.50 | 1.40 | |
