# Supplementary material for: A genome-wide association study of thyroid stimulating hormone and free thyroxine in Danish children and adolescents
Source: PLoS One. 2017 Mar 23;12(3):e0174204. doi: 10.1371/journal.pone.0174204 (PMC5363901; doi:10.1371/journal.pone.0174204)
Supplement: S6 Table — (DOCX) [file pone.0174204.s011.docx]

| **SNP** | **Locus** | **Trait (ranked normalized)** | **Beta in our study (SD)** | **CI_95%_ our study** | **Beta in literature (SD)** | **CI_95%_ in literature** | ***p*** |
| --- | --- | --- | --- | --- | --- | --- | --- |
| rs3008034 | *PDE10A* | TSH | -0.238 | -0.3132;-0.1626 | -0.131 | -0.155;-0.107 | 5.86E-10 |
| rs753760 | *PDE10A* | TSH | 0.238 | 0.1625; 0.3135 | 0.1 | 0.080; 0.120 | 6.43E-10 |
| rs10032216 | *NR3C2* | TSH | 0.238 | 0.1517; 0.3234 | 0.087 | 0.067; 0.107 | 5.92E-08 |
| rs28435578 | *NR3C2* | TSH | -0.229 | -0.3157;-0.1418 | -0.166 | -0.193;-0.139 | 2.50E-07 |
| rs2396084 | *VEGFA* | TSH | -0.197 | -0.2746;-0.1191 | -0.096 | -0.121;-0.071 | 6.91E-07 |
| rs9472138 | *VEGFA* | TSH | -0.19 | -0.2676;-0.1125 | -0.079 | -0.099;-0.059 | 1.56E-06 |
| rs6885099 | *PDE8B* | TSH | -0.118 | -0.1884;-0.0468 | -0.14 | -0.160;-0.120 | 1.13E-03 |
| rs4704397 | *PDE8B* | TSH | 0.117 | 0.0458; 0.1879 | 0.21 | 0.151; 0.269 | 1.27E-03 |
| rs17767742 | *MAF* | TSH | -0.105 | -0.1761;-0.0330 | -0.113 | -0.137;-0.089 | 4.17E-03 |
| rs11624776 | *ITPK1* | TSH | -0.093 | -0.1691;-0.0177 | -0.064 | -0.084;-0.044 | 0.016 |
| rs11755845 | *VEGFA* | TSH | -0.095 | -0.1759;-0.0146 | -0.065 | -0.085;-0.045 | 0.021 |
| rs6923866 | *VEGFA* | TSH | -0.093 | -0.1748;-0.0116 | -0.102 | -0.127;-0.077 | 0.025 |
| rs7568039 | *IGFBP2* | TSH | -0.085 | -0.1618;-0.0091 | -0.122 | -0.149;-0.095 | 0.028 |
| rs116552240 | *ABO* | TSH | 0.068 | -0.0018; 0.1369 | 0.121 | 0.090; 0.152 | 0.056 |
| rs10799824 | *CAPZB* | TSH | -0.084 | -0.1781; 0.0101 | -0.113 | -0.133;-0.093 | 0.080 |
| rs12410532 | *CAPZB* | TSH | -0.083 | -0.1774; 0.0109 | -0.09 | -0.121;-0.059 | 0.083 |
| rs310763 | *SYN2* | TSH | 0.07 | -0.0123; 0.1519 | 0.082 | 0.055; 0.109 | 0.096 |
| rs10519227 | *FGF7* | TSH | -0.06 | -0.1400; 0.0191 | -0.072 | -0.092;-0.052 | 0.14 |
| rs334699 | *NFIA* | TSH | 0.015 | -0.1526; 0.1835 | -0.141 | -0.180;-0.102 | 0.86 |
| rs11726248 | *AADAT* | fT4 | 0.223 | 0.1118; 0.3338 | 0.111 | 0.072; 0.150 | 8.39E-05 |
| rs2235544 | *DIO1* | fT4 | 0.15 | 0.0743; 0.2254 | 0.138 | 0.118; 0.158 | 1.01E-04 |
| rs7694879 | *AADAT* | fT4 | 0.213 | 0.0980; 0.3278 | 0.137 | 0.094; 0.180 | 2.83E-04 |
| rs11103377 | *LHX3* | fT4 | 0.087 | 0.0205; 0.1538 | 0.087 | 0.062; 0.112 | 0.010 |
| rs7860634 | *LHX3* | fT4 | 0.059 | -0.0067; 0.1252 | 0.102 | 0.082; 0.122 | 0.078 |
| rs113107469 | *B4GALT6* | fT4 | 0.118 | -0.1151; 0.3507 | 0.225 | 0.152; 0.298 | 0.32 |
