# Supplementary material for: A genome-wide association study of thyroid stimulating hormone and free thyroxine in Danish children and adolescents
Source: PLoS One. 2017 Mar 23;12(3):e0174204. doi: 10.1371/journal.pone.0174204 (PMC5363901; doi:10.1371/journal.pone.0174204)
Supplement: S7 Table — (DOCX) [file pone.0174204.s012.docx]

| SNP | Proxy | Allele (Effect/Other) |  | Association with obesity | | | Association with BMI SDS | | |
| --- | --- | --- | --- | --- | --- | --- | --- | --- | --- |
|  |  |  | **I^2^** | **OR** | **SE** | **P** | **Beta** | **SE** | **P** |
| rs2983511 | - | C/G | 0.00 | 0.018 | 0.05 | 0.720 | -0.018 | 0.039 | 0.646 |
| rs9968300 | - | A/C | 0.39 | 0.003 | 0.058 | 0.952 | 0.015 | 0.045 | 0.739 |
| rs2396083 | - | C/G | 0.00 | 0.039 | 0.052 | 0.452 | 0.023 | 0.040 | 0.555 |
| rs7847663 | rs925489 | T/C | 0.00 | 0.005 | 0.067 | 0.942 | -0.027 | 0.039 | 0.492 |

S7 Table: Lead SNP’s or proxy’s association to obesity and BMI SDS, using a meta-analysis between the discovery and replication batch.
